# The Korean 3T Practice: New Biosurveillance Model Utilizing New Information Technology and Digital Tools

**DOI:** 10.2196/34284

**Published:** 2022-05-16

**Authors:** HyunJung Kim

**Affiliations:** 1 Barun Information Communications Technology Research Center Yonsei University Seoul Republic of Korea; 2 Department of Biodefense George Mason University Arlington, VA United States

**Keywords:** biodefense, biosurveillance, public health, health security, COVID-19, defense, surveillance, security, South Korea, information technology, digital health, pandemic, testing, tracing, treating, strategy, privacy

## Abstract

In South Korea, COVID-19 pandemic responses, namely the 3T (testing, tracing, and treating) strategy, emerged as a new biosurveillance regime actively using new information technology (IT) and digital tools. The foundation of the Korean 3T system is epidemiological investigation efforts and clinical practices exploiting the use of new digital and IT tools. Due to these unique features, the Korean 3T system can be referred to as a “contact-based biosurveillance system,” which is an advanced version of the traditional biosurveillance models (indicator-based or event-based models). This article illustrates how the contact-based biosurveillance system originated from the experience with the 2015 Middle East Respiratory Syndrome (MERS) outbreak. The post-MERS Korean biosurveillance regime actively adopted the utility of new digital and IT tools to strengthen not only the ex-ante epidemic intelligence capabilities (by traditional models) but also the ex-post response and recovery capabilities (digital contact tracing and digital health intervention). However, critics claim that the Korean 3T system may violate individuals’ privacy and human rights by addressing the fact that the Korean biosurveillance system would strengthen social surveillance and population control by the government as a “digital big brother” in the cyber age. Nevertheless, 3T biosurveillance promises a positive future direction for digital health practice in the current biosurveillance regimes.

## Introduction

The significance of biosurveillance—the real-time pandemic surveillance models enabled by new information technology (IT) and digital tools—emerged following the 2009 H1N1 influenza pandemic [1]. The post-2009 H1N1 biosurveillance regime aimed to strengthen epidemic intelligence capabilities for early warning and timely situation awareness by leveraging new IT and digital tools. Despite the fact that epidemic intelligence integrates and interprets data from both indicator-based and event-based biosurveillance systems, the most recent technological trends in biosurveillance focus primarily on event-based systems [2]. Progress in IT contributes to the development of event-based biosurveillance systems by collecting and monitoring enormous volumes of open internet sources such as news media and social networking services (SNS). While the post-2009 H1N1 biosurveillance regime has highlighted the significance of epidemic intelligence for early warning and timely situation awareness, the world faces the hopeless spread of disease due to an unprecedented pandemic (COVID-19).

Basically, most state-of-the-art surveillance solutions aim to provide intelligence capabilities for either *ex-ante* prevention and preparedness or *ex-post* response and recovery [3]. In particular, the post-2009 H1N1 biosurveillance regime has primarily focused on technological application of epidemic intelligence capabilities for *ex-ante* prevention and preparedness only while downplaying the significance of *ex-post* damage mitigation activities. This article aims to demonstrate that the new Korean biosurveillance regime, which emerged following the 2015 Middle East Respiratory Syndrome (MERS) outbreak, satisfies both the *ex-ante* and *ex-post* biosurveillance objectives during the COVID-19 pandemic. The new coronavirus (SARS-CoV-2) producing the COVID-19 pandemic, like the MERS virus (MERS-CoV), is highly contagious among humans, albeit the novel coronavirus has nonspecific symptoms (flu-like) and asymptomatic transmission. Studies indicate that at least 40% to 50% of those who test positive for COVID-19 exhibit no symptoms [4]. In other words, it was hard to detect and prevent the virus’s influx with flu-like symptoms in the initial phase of the COVID-19 outbreak. As the virus enters and spreads within a community, large-scale testing is essential for successful *ex-post* response and recovery missions.

This article examines how Korea’s past experiences with biosurveillance failures in the 2015 MERS outbreak led to the establishment of the new Korean biosurveillance system capable of conducting both *ex-ante* prevention and *ex-post* response missions. In addition to traditional biosurveillance missions, the post-MERS Korean biosurveillance regime places a strong emphasis on the use of digital and IT technology for *ex-post* response activities, especially digital contract tracing and digital health intervention practices. Unlike other post-2009 H1N1 biosurveillance systems that only focus on *ex-ante* prevention and preparedness efforts (eg, epidemic intelligence), the post-MERS Korean biosurveillance system includes digital contract tracing and digital health intervention practices to respond to and recover from public health emergencies by testing, tracing, and treatment missions. This new system can be referred to as a “contact-based” biosurveillance system because the *ex-post* response activities of the Korean biosurveillance system primarily aim to cut the chain reaction of disease transmission within the community by contact tracing and sending alarms through mobile network systems such as text messages or SNS postings.

## Biosurveillance and New Information Technology

Basically, the biosurveillance regime consists of 2 different systems: event-based and indicator-based surveillance systems. The former model predicts and explains disease outbreaks that could be a serious risk to public health by collecting all reports, stories, rumors, and other information, whereas the latter model is similar to the traditional way of reporting and monitoring clinical cases of specific diseases from hospitals or laboratories [5]. Although technology innovation enables indicator-based biosurveillance systems (or syndromic surveillance) to collect and analyze epidemic data from clinical facilities in near real time, the available literature often emphasizes its complementary role for new event-based systems [6-8].

Scholars praise the increasing role of digital technology and IT that is resulting in the remarkable advance in event-based surveillance systems. New technological advances in information science enhance epidemic intelligence capabilities such as the early warning of infectious disease outbreaks and pandemic situational awareness by collecting voluminous data from multiple internet sources [9]. However, public health groups frequently argue about the existence of huge technological hurdles that new event-based surveillance models should overcome [10,11]. Furthermore, there are inherent concerns about the reliability of sources of event-based epidemic intelligence, which frequently come from various open sources such as news media and SNS as well as official reports from governments and nongovernment organizations [12]. Regardless of these technological limitations and source quantification issues, timeliness is the biggest advantage of new IT-based biosurveillance systems compared with the traditional format of surveillance systems [13]. This is because traditional epidemiology practices are primarily focused on pathogen identification and specific disease ecologies. Thanks to new IT and big data science that improve epidemic intelligence capacities, state-of-the-art biosurveillance systems can develop more reliable predictive models by recognizing and monitoring disease drivers (antecedent conditions) such as climate, weather, war, famine, and human susceptibility to infection [14]. On an international level, advances in digital technology and IT enable international public health regimes (eg, the World Health Organization [WHO]) to establish web-based reporting systems such as the Global Public Health Intelligence Network (GPHIN) based on the International Health Regulation 2005, which enhances the quality of epidemic information and reduces the time it takes to share information among state parties [15].

**Figure 1:**
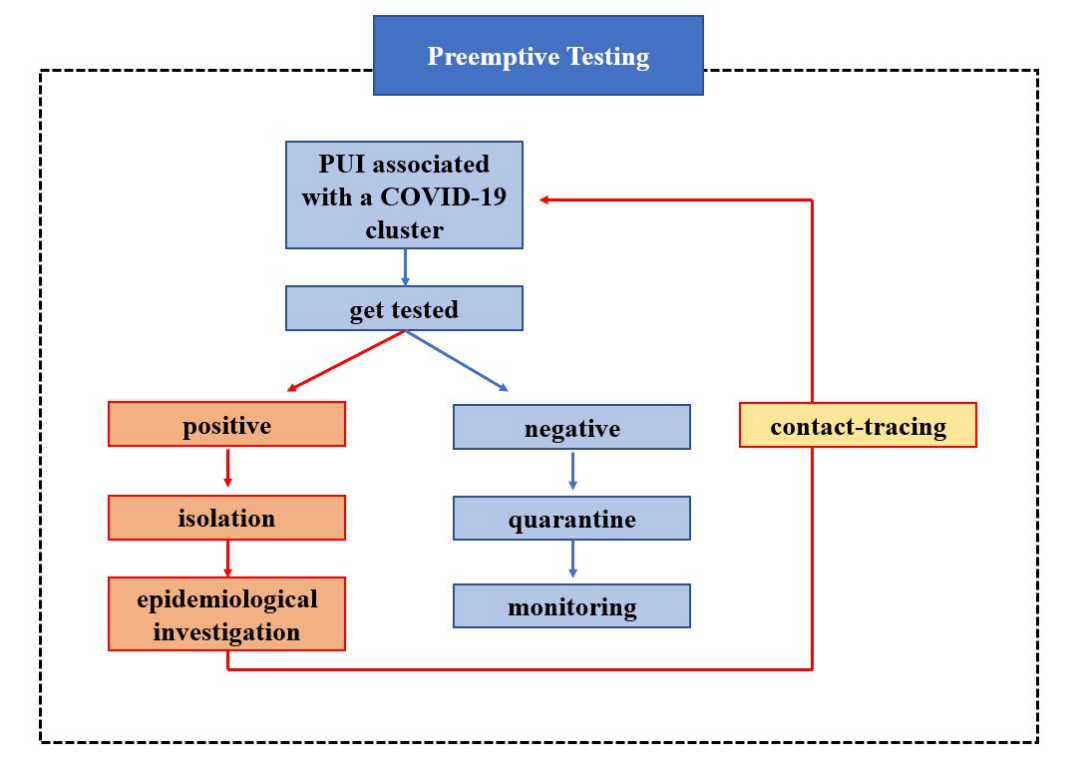
Flow chart depicting the preemptive testing work in the Korean biosurveillance system. PUI: patients under investigation.

Public health expertise aims to enhance epidemic intelligence capabilities for early warning and timely situation awareness by taking advantage of new IT and digital tools. Therefore, the most extensive academic discussions on technology and biosurveillance systems extensively focus on event-based biosurveillance models when considering how to provide more timely, reliable, and accurate epidemic intelligence. However, following the COVID-19 pandemic outbreaks throughout the world, event-based biosurveillance models have proven less effective for disease control and prevention efforts. Flu-like symptoms caused by the coronavirus often make it difficult to identify patients quickly [16]. US public health authorities have no option but to encourage people to “Stay Home When You Are Sick” [17]. In response to this grim reality, South Korea introduced a new form of biosurveillance that demonstrates more effective disease control and prevention performances, namely the 3T practice, comprising testing, tracing, and treatment [18]. Unlike most event-based biosurveillance systems that focus on early warning and situation awareness capabilities, the Korean 3T biosurveillance employs an “Active Search strategy,” which aims to actively search and trace all suspected cases that may have had close contact with confirmed cases through preemptive testing practices [19]. All confirmed cases, as presented in Figure 1, should be isolated. Based on the mobility history of confirmed cases, all suspected cases who may have had close contact with or are patients under investigation (PUI) associated with a COVID-19 cluster should go to public health centers for preemptive testing.

## Failed Biosurveillance: Lessons Learned From the 2015 MERS Outbreak

To better understand the features and origins of the contact-based surveillance system in South Korea, it is necessary to examine what is considered a “focusing event” and how this system has evolved. The concept of the focusing event often accounts for the origin of institutional changes in the language of *critical juncture*, which is a decisive moment of innovation caused by crises (exogenous shocks) such as a revolution, war, or regime change [20-23]. Disasters (eg, pandemic or 9/11 terrorism) often provide lessons that a country can learn from; these disasters are *focusing events* that lead to the adoption of new policies due to the increased attention on a new agenda and for the mobilization of interest groups [24,25]. Indeed, after the 2015 MERS outbreak, disease containment and epidemiology became the center of the public health policy agenda in Korea, which highlights nonpharmaceutical interventions in considering how to build effective “diagnose and detect” capabilities to break the chain reaction of infectious disease transmissions [26]. Therefore, the lessons learned from the 2015 MERS experiences affected all public health–related areas in Korea and laid the groundwork for the institutionalization of the post-MERS Korea public health systems to function efficiently during the COVID-19 pandemic [27].

The 2015 MERS outbreak facilitated a major revision of the biosurveillance regime in South Korea due to the biosurveillance failures. The first lesson that the Koreans gained from the MERS outbreak was to recognize the flaws of the event-based system. Although the event-based biosurveillance system is technologically advanced, it contains a loophole that allows the inflow of infectious diseases. The WHO delivered a MERS-related epidemic advisory via the GPHIN, a secure Internet-based multilingual early warning tool developed by Health Canada in collaboration with the WHO [28]. Based on GPHIN sources, the South Korean government did not include Bahrain as a MERS-dangerous zone because, despite its geographical proximity to Saudi Arabia, the country with the largest MERS outbreak, no cases were initially reported in Bahrain. Indeed, the first case of MERS infection was reported in Bahrain on April 10, 2016 [29]. In May 2015, when a sick businessman (patient zero) sought medical treatment for a high fever and other flu-like symptoms, the South Korean public health authorities ignored the possibility that the patient, who had recently returned from Bahrain, may have been infected with MERS. Even though patient zero had visited Saudi Arabia, the country with the MERS outbreak, no public health system could track his travel history. Since patient zero entered Korea through a breach in the Korean biosurveillance system relying on the GPHIN, almost 2 weeks had elapsed before he was officially confirmed to be infected with MERS on May 20, 2020.

The second lesson is that, during highly infectious disease outbreaks, the traditional model of an indicator-based surveillance system could not work for disease control and prevention practices. While relying on the international event-based biosurveillance system (the GPHIN) as the primary source of epidemic intelligence, the Korean public health authority has adhered to the traditional indicator-based models. However, when medical and health care systems were overloaded or damaged due to uncontrollable disease spread, the indicator-based systems reporting cases from health care providers, physicians, or laboratories were completely dysfunctional. For 2 weeks, patient zero visited 3 different hospitals before finally being admitted to the Samsung General Hospital, infecting 82 other people. MERS-CoV, the virus that causes MERS, is a member of the *coronaviridae* family, which amplified nosocomial infection within hospitals during the MERS outbreak in Korea [30]. In general, hospitals are hubs for sick people who are vulnerable to any kind of contagious disease. Due to the nosocomial feature of the MERS virus, hospitals unwittingly became major sites for MERS transmission. For example, St. Mary’s Hospital in Pyeongtaek, 1 of the 3 hospitals visited by patient zero, became the most notorious virus breeding spot because 28 people were infected.

Since the MERS outbreak emasculated both the biosurveillance systems, a super-spreader issue was highlighted in Korean society. A super-spreader is someone who, before being confirmed with MERS infection, had spread the disease to many other people, exacerbating the uncontrollable chain reaction of disease transmissions. Patients 0, 14, and 16 were labeled as super-spreaders [31]. Patient zero initiated a chain reaction of illness transmission in numerous institutions by unwittingly infecting so many health care workers and patients. Patient 14, a secondary infection from patient zero, also visited the Samsung General Hospital, which resulted in 85 cases. Patient 16, another secondary infection from patient zero, infected 23 people in other hospitals. The super-spreaders were not only staying at hospitals but also freely walking down streets. The mild flu-like symptoms in the early phase of the MERS infection made disease control and diagnosis much harder. During the MERS outbreak, no one knew who was infected or which hospitals were contaminated. The super-spreader issues increased public fear of possible contact with confirmed cases or unknown carriers in any public space, aggravating social chaos in Korea.

## Application of Digital and IT Tools to New Contact-Based Biosurveillance

Since the MERS outbreak, the Korean public health authority has realized the significance of upgrading the public health surveillance system to respond to public health emergencies. It is especially necessary to strengthen real-time epidemic information-sharing capabilities among international and domestic stakeholders and increase interagency communication capabilities in the post-MERS biosurveillance regime [32]. Following MERS, the Korean biosurveillance regime began to strengthen domestic event-based surveillance systems for early warning and timely threat awareness, which can complement the limitations of the established indicator-based surveillance system [33]. In addition to early warning surveillance capabilities, the new post-MERS biosurveillance regime aims to strengthen the rapid implementation of control measures (*ex-post* intervention for response and recovery) through rigorous epidemiological investigations, contributing to the successful defense of the MERS inflow in 2018 [34].

**Figure 2:**
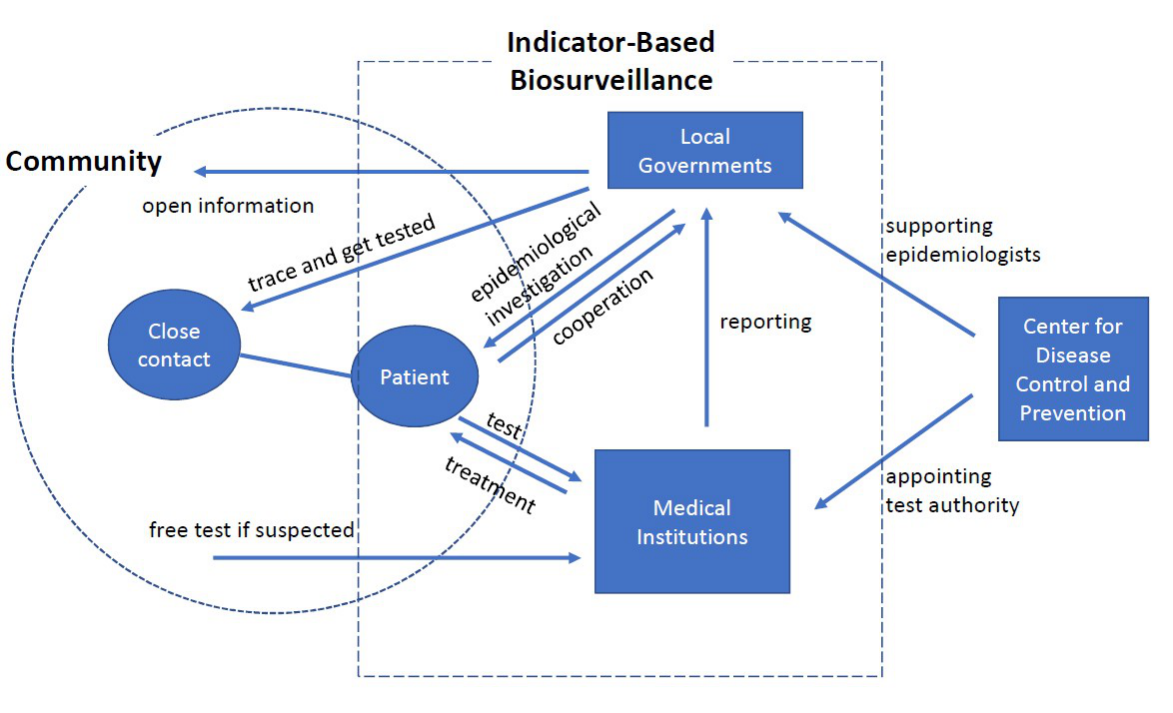
The extended operation mechanism of the Korean biosurveillance system [35]. Govts: governments.

Interestingly, closer scrutiny reveals that the post-MERS Korean biosurveillance implementation of control measures seems like an extended version of the traditional indicator-based biosurveillance system. As presented in Figure 2, the operational mechanism of post-MERS Korean biosurveillance is similar to indicator-based models (within the dotted-line box), although it performs more extensive activities. When a patient is diagnosed with a disease, medical institutions (eg, public health centers) conducting polymerase chain reaction (PCR) tests immediately report the testing result to the local government that has jurisdiction over the patient’s address. However, the differences from other indicator-based systems start from epidemiological investigations.

A prominent feature of the post-MERS biosurveillance system is digital contact tracing by epidemiological investigations utilizing new digital and IT tools. In contrast to other biosurveillance models utilizing digital and IT tools for developing computerized predictive models, new IT and digital tools are mainly exploited for epidemiological investigation in the Korean biosurveillance system. It is because the post-MERS Korean biosurveillance regime institutionalizes the use of new digital and IT platforms such as GPS tracking to strengthen contact tracing capabilities, thus preventing unknown routes of disease transmission from super-spreaders. To foster the new biosurveillance regime, the Infectious Disease Control and Prevention Act, often called the MERS Act, was enforced in January 2016 as follows [36]:

...the Minister of Health and Welfare shall promptly disclose information with which citizens are required to be acquainted for preventing the infectious disease, such as the movement paths, transportation means, medical treatment institutions, and contacts of patients of the infectious disease.

The Republic of Korea, Article 34-2

Article 34-2 of the MERS Act establishes a legal basis for digital contact tracing practices, allowing the government to utilize all possible digital and IT resources to trace all PUIs through epidemiological investigations. This legislative effort allows the local governments to document the mobility history of the patients down to the minute based on a comprehensive epidemiological investigation through testimony, closed-circuit television (CCTV), smartphone GPS, and credit card transactions. Digital contact tracing practices—epidemiological investigation utilizing different digital and IT resources—can contribute to the timely collection of epidemic information to search for all PUIs.

Another outstanding feature of the new Korean biosurveillance system is the utility of digital and IT tools for active interventions when the government performs *ex-post* response and recovery missions during a public health emergency. First, since they collect all epidemic information through digital contact tracing practices, the local governments have released all information on the movements of the patients to the public by text messages and SNS postings, including where they went, when they were there, and how they got there. Because the epidemic information was made public, other people in the same community could avoid the areas the patients had visited. Furthermore, the public disclosure may encourage people who have visited those places at the same time to seek medical attention as soon as possible and to enter self-quarantine if they have similar symptoms. Second, based on the collected epidemic information from epidemiological investigations, local governments can identify and trace all PUIs and notify them via text messages that they need to get tested. All PUIs who have received a text message are required to visit public health centers and get tested. Public disclosure of epidemic information and notices or alarms sent via text messages to get tested are forms of mobile health (mHealth) practices, which is one of the main pillars of digital health intervention.

Digital health is an emerging concept and a new form of medical practice described as “the broad scope of digital health that includes categories such as mHealth, health IT, wearable devices, telehealth and telemedicine, and personalized medicines” [37]. Disease diagnosis by new digital health is the most applicable component of digital health, and medical artificial intelligence has been highlighted as the future direction of digital health in clinical practice [38]. In this vein, the Korean 3T biosurveillance system, exploiting new digital and IT tools for digital contract tracing practices (identifying and tracing all PUIs) and for intervening in clinical practice (notices to all PUIs getting tested), is more than just a biosurveillance regime in general. The 3T surveillance works for national-level digital health intervention practices for *ex-post* response and recovery missions during a public health emergency, beyond the *ex-ante* prevention missions by traditional event-based and indicator-based biosurveillance models.

## Limitations and Side Effects

The Korean 3T practice is a new type of biosurveillance model—a contact-based model—which actively uses digital and IT tools to identify and trace all PUIs. Furthermore, this model is tailored for digital health intervention practices by requiring PUIs to be diagnosed and treated, which can help break the chain reaction of disease transmission within communities. Despite its success in disease control and preventive techniques, the contact-based biosurveillance system is plagued by privacy and human rights violations. For example, basic personal information, including age, gender, and place of residence, is made public. When collecting and disclosing their personal information, patients’ consent was not required. Patients must cooperate with public health authorities during the epidemiological investigation phase; if they do not cooperate or give false information, patients are subject to punishment under the law. In addition, a person’s travel history, describing when he or she arrived at X and moved to Y and stopped by Z, is documented down to the minute; for example, the person entered a restroom at 18:05, left at 18:08, walked from C to D, drove their car in front of E library, arrived home at 18:16. In some cases, it is not difficult to figure out who he or she is. Due to such detailed private information, several media outlets warn that Korean biosurveillance risks violating the privacy and human rights of citizens [39,40]. Scholars are doubtful that the United States and other developed countries can adopt such an aggressive digital contract tracing practice because they are worried about the loss of privacy and civil liberties [41,42]. Later, in June 2020, as a result of acrid debates in terms of privacy issues, the Korean government released a rigid “guideline for public disclosure,” which aims to protect patients from unwanted exposure of their personal identity and privacy. Local governments should provide just the minimum information essential for public health missions, such as guidelines requiring no home address and no age, sex, nor nationality.

Contact-based biosurveillance systems, in particular those that rely on epidemiological investigation and tracing all PUIs, are harsh for minority groups, and those who intentionally violate the consensus of the community abiding by the biosurveillance regime become a target for normative criticisms. The Itaewon case exemplifies the dark side of the Korean surveillance regime. Itaewon is an international district of Seoul, the capital city of South Korea, that symbolizes freedom and liberation for young people. Unsurprisingly, many famous clubs in the LGBT community are located in the Itaewon district, and one of them became a hotspot for the COVID-19 outbreak. News media outlets were scrambling to report the new possible pandemic wave, using an incendiary and pejorative term—Gay Club—which sparked a huge backlash against the Korean LGBT community [43]. People at the club were worried about their sexual identities being revealed. They attempted to avoid the government’s testing guidelines to conceal their identities, thus leaving 5000 people uncontactable [44]. The Korean government finally decided to adopt the most aggressive measures to trace these people. The government worked with telecom carriers to determine who accessed the Itaewon cell towers at the time to trace down the people who were in Itaewon [45]. Consequently, the people listed as wireless providers stand at the center of public outrage and have even been accused by the public health authorities.

## Conclusion

Both event-based and indicator-based biosurveillance systems offer remarkable predictive public health models that work for effective disease control and prevention practices. Much research has been conducted on the application of new IT and digital tools to enhance the reliability and accuracy of biosurveillance systems. Since the COVID-19 outbreak, the world has realized that current biosurveillance systems are ineffective in dealing with the unprecedented pandemic. In particular, the spread of the novel coronavirus, which is highly contagious but has no unique symptoms, is hardly detectable and traceable within communities. When responding to the COVID-19 outbreak, however, the 3T practices of South Korea can present a new biosurveillance model exploiting new IT and digital tools in the cyber age. This Korean biosurveillance system is specialized in digital contract tracing practices that conduct epidemiological investigations on all close-contact people through new IT and digital tools. As the epidemic information (eg, a patient’s travel history) is acquired by CCTV or other digital resources and is disclosed to the public, people who have been to those places at the same time can seek medical attention quickly and be tested. Based on the collected epidemic information, the Korean public health authority identifies and traces those who may have been in close contact with patients and reaches out to all PUI to ensure they are tested. This biosurveillance system, consisting of test, trace, and treatment practices enabled by the new digital and IT tools, performs well when it comes to breaking the chain reaction of disease transmission within a community.

In other words, the conventional models of both event-based and indicator-based biosurveillance systems optimize the epidemic intelligence operation that predicts the potential and actual infectious disease outbreaks only during prepandemic conditions, while the 3T practice works for *ex-post* response missions such as digital contact tracing and digital health intervention practices during an ongoing pandemic. Despite these merits, it is necessary to caution people about the side effects of the Korea 3T biosurveillance system. Korean public health authorities should be aware of the potential risks of violating human rights and privacy when operating contact-based biosurveillance. Therefore, it is necessary to develop complementary measures that can close the gaps of 3T practice.

## Abbreviations

3T: testing, tracing, and treating
CCTV: closed-circuit television
GPHIN: Global Public Health Intelligence Network
IT: information technology
MERS: Middle East Respiratory Syndrome
mHealth: mobile health
PCR: polymerase chain reaction
PUI: patients under investigation
SNS: social networking services
WHO: World Health Organization
